# Reference intervals for knee functions specific to outpatients with knee osteoarthritis: a cross-sectional study

**DOI:** 10.1186/s13102-024-00901-w

**Published:** 2024-05-18

**Authors:** Hideyuki Ito, Tetsuya Amano, Kiyoshi Ichihara

**Affiliations:** 1 Department of Rehabilitation, Faculty of Wakayama Health Care Science, Takarazuka University of Medical and Health Care, 2252 Nakanoshima, Wakayama, Wakayama-Pref.640-8392 Japan; 2 Department of Physical Therapy, Faculty of Health and Medical Sciences, Tokoha University, Hamamatsu, Japan; 3 Faculty of Health Sciences, Yamaguchi University Graduate School of Medicine, Ube, Japan

**Keywords:** Reference value, Knee osteoarthritis, Muscle strength, Range of motion

## Abstract

**Background:**

Reference values (RVs) for knee function tests have been reported in perioperative patients with knee osteoarthritis (KOA); however, such values for practical use in outpatient setting has yet to be determined. Therefore, we aimed to establish the reference intervals (RIs) for outpatients with mild to moderate KOA.

**Methods:**

This cross-sectional study enrolled 202 outpatients with KOA from 8 Japanese orthopedic clinics and measured knee extensor/flexor muscle strength (MS) and knee extension/flexion range of motion (ROM). We used multiple regression analysis to evaluate the sources of variation, including sex, age, body mass index, Kellgren–Lawrence (K-L) classification, bilateral KOA, and exercise habits. Magnitude of between-subgroup differences is expressed as standard deviation ratio (SDR) based on a three-level nested analysis of variance, with SDR ≥ 0.4 as the threshold for requiring RIs specific for subgroups. RIs were calculated parametrically using two-parameter Box-Cox formula if Gaussian transformation of RVs was successful, otherwise calculated nonparametrically.

**Results:**

Partitioning was required by sex for extensor and flexor MS (SDR = 0.65, 0.57, respectively) and by K-L classification for flexion ROM (SDR = 0.54). RIs were determined parametrically for extensor MS as 0.27–2.09 (male) and 0.27–1.54 (female) Nm/kg and for flexor MS 0.18–1.20 (male) and 0.13–0.79 (female) Nm/kg. On the other hand, RIs for extension and flexion ROM were determined nonparametrically due to discrete nature of their RVs. The RIs determined for extension ROM were -15°–0° and for flexion ROM were 105°–150° (for K-L grade I/II) and 95°–140° (for K-L grade III/IV).

**Conclusions:**

The ranges of RIs determined specifically for patients with mild to moderate KOA were in-between those of age-matched healthy controls and pre-surgical KOA patients, both of which we had reported for use in physiotherapeutic management of KOA patients undergone total knee arthroplasty. The newly derived RIs will provide an objective benchmark for physiotherapy targeting outpatients with mild to moderate KOA.

## Background

The large cohort study of Japanese population reported that the prevalence of knee osteoarthritis (KOA) was 42.6% for men and 62.4% for women among residents aged 40 years or older [1]. Knee joint function is often impaired in patients with KOA, especially knee muscle strength and range of motion (ROM), which affect activities of daily living and quality of life [2, 3]. Obviously, KOA is the most prevalent and important target disease in the field of rehabilitation medicine, and, according to Japanese Physiotherapy Guidelines [4], physical therapy such as quadriceps muscle strengthening exercises and ROM exercises are primarily indicated for KOA patients depending on the degree of reduction in the motor function.

Standard physical therapy, including muscle strengthening and joint mobilization exercises have been reported to improve activities of daily living [5, 6]. To determine the indication for standard physical therapy, the patients must be evaluated for muscle weakness and limited ROM. It is then important to evaluate the levels of motor functions of KOA patients based on their reference intervals (RIs), which shoud be established from the KOA outpatients.

To read the motor function test results objectively, it is essential to understand their sources of variation (SV) and to have a reliable RIs for each parameter [7, 8].

Previous studies have reported RIs that were set for healthy participants [9–11] and perioperative KOA patients [11]. However, those RIs for healthy subjects and perioperative KOA patients should not match to the RIs of KOA outpatients. In addition, establishment of severity-specific RIs should be required for use in tailoring the musculoskeletal rehabilitation for preventing the progression of KOA.

Based on these backgrounds, we conducted a multicenter study to evaluate SVs of knee functions and to establish SV-based stratified RIs for use in objective management of KOA outpatients undergoing musculoskeletal rehabilitation.

## Methods

### Study design and participants

The study design was a cross-sectional study aimed at establishing RIs of muscle strength and ROM in outpatients with KOA. The study recruited 218 outpatients with KOA from eight Japanese orthopedic clinic between August 2018 and January 2023. The inclusion criteria were outpatients 1) diagnosed with KOA by physicians and 2) who provided written consent. The exclusion criteria were 1) the presence of motor paralysis or other neurological dysfunctions such as stroke, lumbar disc herniation, spinal canal stenosis, or peripheral neuropathy due to diabetes mellitus unrelated to KOA; 2) obvious motion pain or restricted joint movement in locations other than the knee, which hinders walking or sit-to-stand motion; 3) cognitive impairment; and 4) missing motor function test results. Accordingly, three patients with stroke, eight with neurological impairment related to spondylolisthesis, and five with apparent movement limitation for reasons other than the knee were excluded from the study. Finally, 202 patients were included for subsequent analyses. For comparison, we included RIs determined in our previous study from 545 advanced KOA patients just prior to total knee arthroplasty and from 120 age-matched healthy subjects [11].

## Measurements

### Basic physical and medical attributes

We recorded information regarding sex, age, body mass index (BMI), and Kellgren–Lawrence (K-L) classification [12] to assess the severity of knee joint degeneration, affected sides (unilateral or bilateral), and regular exercise habits (defined as exercise performed at least twice weekly for 30 min or more).

### Knee muscle strength

Extensor muscle strength was measured using a handheld dynamometer (HHD) (μTas F-1; Anima Corp, Tokyo, Japan) according to the following procedure. The subject was placed in the sitting position with his lower limbs fixed with a belt so that the knee joint was in a 90° flexion position. Torque (Nm), the product of HHD output (N) and arm length (m), was calculated, and the torque-to-weight ratio (Nm/kg), calculated by dividing the value by the subject's body weight (kg), was used as the muscle strength value. The arm length was measured from the lateral cleft of the knee joint to the center of the sensorium on the front surface of the distal lower leg [13, 14]. Muscle strength (Nm/kg) was expressed as torque divided by body weight (kg) and measured twice; the average value was used because the variability in measurement was higher in older women [15, 16].

Knee flexor muscle strength was measured in the sitting position with arms crossed in front of the chest and trunk and pelvis in the middle position. The examiner stood in front of the subject side and fixed the subject side with a belt so that the knee joint was in a 90° flexion position. The belt was fixed to the examiner's distal lower leg for measurement. Knee flexor muscle strength was also divided by the subject's body weight (kg), and the torque-to-weight ratio (Nm/kg) was used as the muscle force value. The maximum isometric muscle force of each muscle was measured twice, and the average value was adopted [13, 14].

### Measurement of knee ROM

The ROM during flexion and extension of the knee joint was measured using a joint goniometer in accordance with the guidelines jointly published by the Japan Orthopedic Surgery Society and Japan Rehabilitation Medical Society [17]. The angle between the long axis of the tibia (on the line connecting the fibula head and lateral ankle) and femur (on the line connecting the greater trochanter and lateral femoral epicondyle) was measured in 5° increments.

## Statistical methods

### SVs of motor function parameters

Multiple regression analysis was performed to identify factors associated with exercise function parameters. The following parameters were evaluated as explanatory variables and used in the regression model: gender, age, BMI, regular exercise habits (binary: no = 0, yes = 1), K-L classification (dummy variables were set for K-L grades II, III and IV by setting K-L grade I as the reference category). All explanatory variables were sex fixed in the regression model for all dependent variables to facilitate comparisons among parameters. The practical level of significance of each standardized partial regression coefficient (r_p_) was expressed by using a threshold of |r_p_ |≥ 0.2, corresponding to the midpoint Cohen's effect size small (0.1) and medium (0.3) of the correlation coefficient [18].

For knee extensor and flexor muscle strength, a three-level nested analysis of variance (ANOVA) was performed with sex, age, and BMI as the three factors. On the other hand, for knee flexion and extension ROM, ANOVA was performed with factors set for sex, age, and K-L classification. The three factors were selected based on the results of multiple regression analysis. In the analysis, age and BMI were converted to a rank scale by dividing the values at 70 and 80 years of age (3 categories) and BMI at 18.5, 25, and 30 kg/m2 (based on 4 categories of World Health Organization (WHO) standards).

The analysis provided the magnitude of each SV in terms of SD: SD for sex (SDsex), age (SDage), BMI (SDbmi), K-L classification (SDk-l), and between-individual SD (SDindiv). The relative magnitude of each SD was expressed by calculating its ratio to SDindiv. We designated this as the SD ratio (SDR): SDRsex = SDsex/SDindiv; SDRage = SDage/SDindiv; SDRbmi = SDbmi/SDindiv; and SDRk-l = SDk-l/SDindiv. The threshold value for the SDR at which we chose to partition test results by the factor was set to 0.4, as previously reported [7, 8].

### Method for statistical derivation of RIs

The RI of the motor function parameters was derived using the following two-parameter Boc-Cox equation [19], a parametric method that features a power transformation of the values (test results) to make their distribution Gaussian.

$$X=\frac{{(x-a)}^p-1}p,$$

Where X represents the transformed value of the test result *x*; *p* and *a* represent the power and origin of the transformation, respectively. The central 95% interval under the transformed scale (LL^T^, UL^T^) was calculated using the mean and SD of the transformed test results (m^T^, SD^T^) as follows:

$$\mathrm{LL}^{\mathrm T}=\mathrm m^{\mathrm T}-1.96\;\mathrm{SD}^{\mathrm T}$$

$$\mathrm{UL}^{\mathrm T}=\mathrm m^{\mathrm T}+1.96\;\mathrm{SD}^{\mathrm T}$$

The lower and upper limits (LL and UL) in the original scale were then calculated by inverse transforming using the following equations:

$$\mathrm{LL}\;=\;{(\mathrm p\;\times\;\mathrm{LL}^{\mathrm T}+\;1)}^{1/\mathrm p}+\;\mathrm a$$

$$\mathrm{UL}\;=\;\left(\mathrm p\;\times\;\mathrm{UL}^{\mathrm T}\;+1\right)^{1/\mathrm p}+\;\mathrm a$$

The success of the Gaussian transform was confirmed by the linearity of the cumulative frequency curve on the probability plot and the Kolmogorov–Smirnov test of normality.

If given test results failed to attain Gaussian distribution, or if the test results had discrete values, the nonparametric method was applied, and the central 95% range of the test results was calculated as 2.5th and 97.5th percentiles.

## Results

### Profiles of KOA patients

Our participants comprised 43 males and 159 females (Table 1). Regular exercise habits were self-reported as “yes” by 71 and “no” by 131 patients. The knee joints were affected unilaterally in 110 patients and bilaterally in 92 patients. According to the K-L classification, the severity of knee joint deformity was grade I in 45, II in 67, III in 68, and IV in 22 patients.

**Table 1:** Demography of KOA patients and healthy volunteers

	KOA-Outpatients				Healthy volunteers		KOA-PreSurg		
Sex(n)	M:43 F:159				M:36 F:84		M:127 F:418		
Age(year)	72.6 ± 9.2				71.3 ± 5.9		74.2 ± 7.7		
BMI(kg/m^2^)	24.4 ± 3.9				22.5 ± 2.9		25.3 ± 3.7		
Exercise(n)	yes:71 no:131				yes:99 no:21		yes:176 no:369		
K-Lgrade(n)	I:45	II:67	III:68	IV:22			II:29	III:252	IV:264
Affected knee(n)	unilateral:110 bilateral:92						unilateral:205 bilateral:340		

*KOA* Knee osteoarthritis, *BMI* Body Mass Index, *K-L* Kellgren-Lawrence

### SVs of knee functions

Multiple regression analysis revealed that sex was a significant SV with |rp|> 0.2 as indicated by rp values of -0.36 and -0.32 for extensor and flexor muscle strength, respectively. Age was negatively associated with flexor muscle strength (rp = -0.20), whereas BMI was negatively associated with extensor (rp = -0.28) and flexor muscle strength (rp = -0.23). Compared with K-L grade I, K-L grades II and IV were negatively associated with flexion ROM (rp = -0.21 and -0.39, respectively), whereas K-L grade III was negatively associated with extension and flexion ROM (rp = -0.40 and -0.48, respectively) (Table 2).

**Table 2:** Multiple regression analysis for sources of variation of knee function tests

	**R**	**Sex**		**Age**		**BMI**		**K-L** **gr II**		**K-L** **gr III**		**K-L** **gr IV**		**Bilateral**	**Exercise**
Extensor MS	0.491	**-0.357**	***	**-0.174**	*	**-0.284**	**	-0.107		-0.153		-0.035		0.076	0.062
Flexor MS	0.456	**-0.317**	***	**-0.200**	*	**-0.234**	*	**-0.178**	*	-0.130		-0.091		0.123	0.060
Extension ROM	0.477	**-0.164**	*	**-0.190**	*	-0.093		-0.131		**-0.396**	***	**-0.193**	*	-0.072	-0.007
Flexion ROM	0.477	-0.004		-0.056		-0.134		**-0.208**	*	**-0.478**	***	**-0.394**	***	0.073	0.010

Multiple regression analysis was performed by setting each motor function parameter as a dependent variable and a fixed list of explanatory variables: sex, age, BMI, exercise, K-L grade, and bilateral. The dummy variables for K-L grades were made by setting K-L grade = 1 as a reference category. The values listed are standardized partial regression coefficients (r_p_), which take values between -1.0 and 1.0. The values above the effect size of |r_p_|≥ 0.2 are highlighted in bold letters. Statistical significance is indicated as *P < 0.01, **P < 0.001, and ***P < 0.0001

*R* Multiple correlation coefficient, *BMI* Body mass index, *K-L* Kellgren–Lawrence (ordinal) from 2 to 4, *Bilateral* (binary) indicating bilateral osteoarthritis involvement of knees, *Exercise* (binary) indicates regular exercise habit

**Fig. 1:**
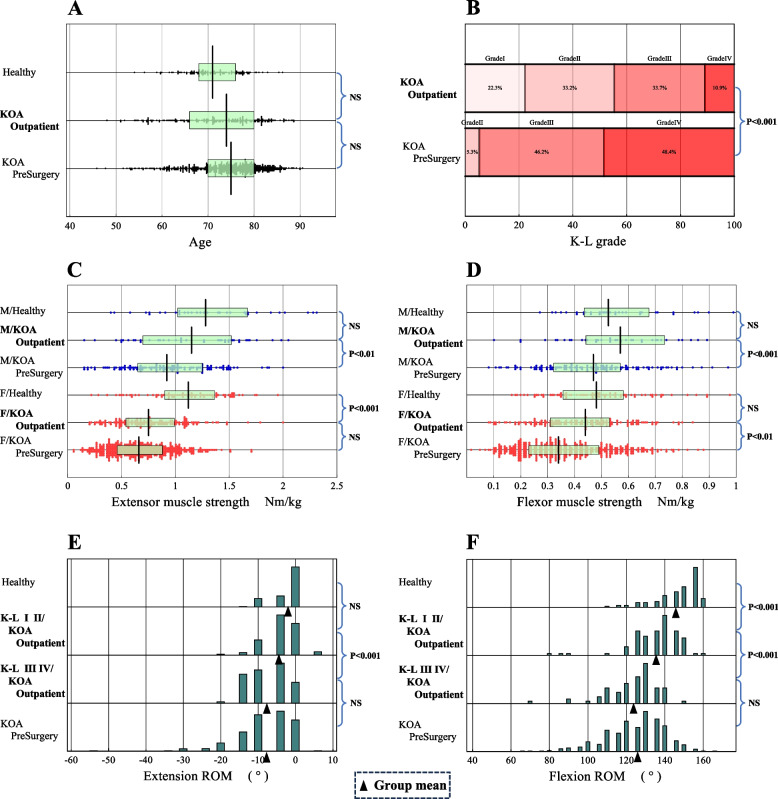
Comparison of demographic information and motor functions across three groups. Comparison of demographic information and motor functions across three groups: healthy controls, KOA outpatients, and preoperative KOA patients just prior to total knee arthroplasty [11]. The distribution of flexion ROM test results for KOA outpatients was further partitioned by the K-L grade (I/II and III/IV). In 1**C** and 1**D**, the box and its central vertical bar drawn overlaid in each scattergram represents central 50% range and the median, respectively. In 1**E** and 1**F**, the triangle symbol indicates a location of a mean of ROM values for each subgroup

The extensor and flexor muscle strengths (MS) were compared in Fig. 1C and 1D after partitioning by sex. Extensor and flexor muscle strength was lower in the women. The knee extensor MSs of outpatients were in the middle location between healthy controls and KOA preoperative patients. The knee extensors MS of male patients differed from those of preoperative patients (Fig. 1C), and those of female patients differed from those of healthy controls. The flexor MS of outpatients differed from those of preoperative patients in both sexes (Fig. 1D).

As illustrated in Fig. 1E and 1F, ROMs of the outpatients were intermediate between those of the healthy controls and the preoperative patients. In addition, significant differences in knee joint extension ROM were observed according to the K-L grade between I/ II and III/IV. On the other hand, significant differences in knee flexion ROM were observed between healthy subjects and K-L I/II outpatients, and also between K-L I/II and K-L III/IV. It is of note that the changes in flexion ROM by K-L grade were more prominent than that of extension ROM.

### Assessment of factors to derive RIs specific to subgroups

We considered sex, age, BMI, and K-L grade as possible factors requiring subgrouping of the test results when deriving RIs. As presented in Table 3, by setting SDR ≥ 0.4 as the threshold for the values, partitioning by sex was required for extensor and flexor muscle strength, with SDRsex of 0.652 and 0.567, respectively. In contrast, for knee flexion ROM, partitioning by the K-L grade was required (SDRk-l = 0.538). Dividing the RI into four categories (K-L grades I–IV) would make the RI unstable because of the small sample size. Therefore, the RI was divided into two categories, with the boundary between K-L II and K-L III, because the difference in rp values between K-L II (rp = -0.21) and K-L III (rp = -0.48) relative to K-LI was significant. As the SDRs for extension ROM were all below the threshold, ROM values partitioning by sex, age, or K-L grade was not required.

**Table 3:** The list of SDRs calculated for assessing the need for partitioning values

	**SDRsex**	**SDRage**	**SDR**_**BMI**_	**SDRK-L**
Extensor muscle strength	**0.652**	0.000	0.339	−
Flexor muscle strength	**0.567**	0.000	0.352	−
Extension ROM	0.291	0.207	−	0.380
Flexion ROM	0.000	0.000	−	**0.538**

Three-level nested ANOVA was performed in two ways. That for muscle strength was performed by setting sex, and age, and BMI as factors of variations, and that for ROM was performed by setting sex, and age, and K-L grade as the factors. In the analyses, age and BMI were respectively partitioned at 70 and 80 years and 18.5, 25, and 30 kg/m2. We regard SDR ≥ 0.4 as a guide to judge the need for partitioning test results into subgroups in deriving the RIs

### Determination of RIs

**Fig. 2:**
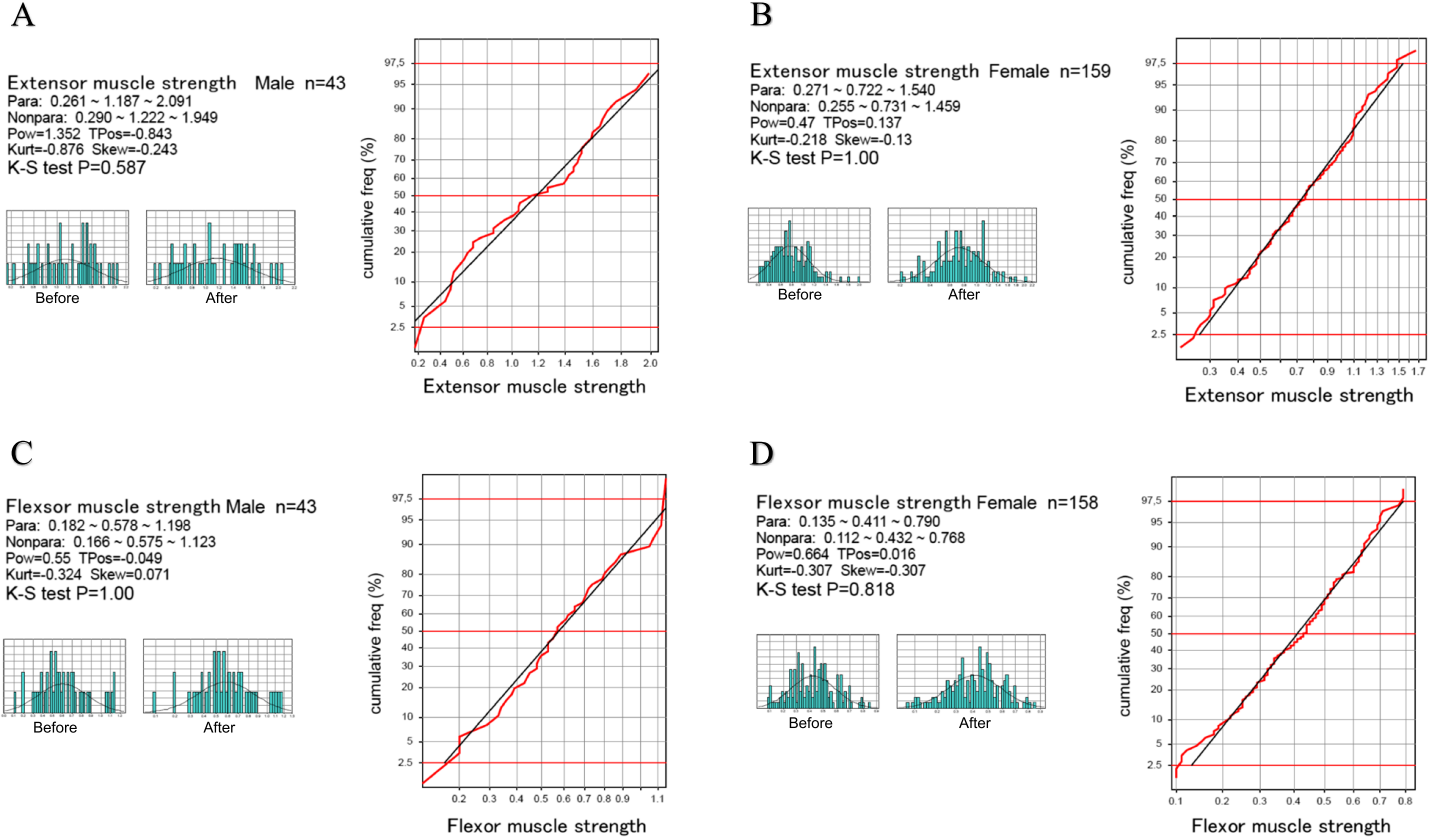
Confirmation of normalization of the distribution of muscle strength using the parametric method. The distribution of extensor and flexor muscle strength test results are shown by histograms before and after Gaussian transformation (on the left) and by the probability plot (on the right) to demonstrate the validity of using the parametric method based on the two-parameter Box-Cox power transformation formula (see the main text) [19] Para: RI by parametric method (LL~Me~UL); Nonpara: RI by nonparametric method; Pow: power; TPos: transformation origin or shift location; Kurt: kurtosis; Skew: skewness; K-S test: Kolomogorov-Smirnov test

The RIs for knee function test results were derived guided by the SDR values (Table 4). With confirmation of successful Gaussian transformation by Kolmogorov–Smirnov test (Fig. 2), the RIs for extensor and flexor MSs were determined by the parametric method. While the nonparametric method of was used to determine the RIs for knee extension and flexion ROMs because their angle values were recorded discretely at every 5°.

**Table 4:** The list of reference intervals derived for knee motor functions

**Extensor muscle strength**					
	parametric RIs				
	Sex	n	LL	Me	UL
Healthy^*1^	All	120	0.37	1.16	2.00
KOA-Outpatient	All	202	0.27	0.78	1.83
	M	43	0.27	1.19	2.09
	F	159	0.27	0.72	1.54
KOA-PreSurgery^*2^	M	124	0.25	0.91	1.88
	F	404	0.20	0.65	1.36
**Flexor muscle strength**					
	parametric RIs				
	Sex	n	LL	Me	UL
Healthy*1	All	120	0.21	0.49	0.93
KOA-Outpatient	All	201	0.13	0.44	0.91
	M	43	0.18	0.58	1.20
	F	158	0.13	0.41	0.79
KOA-PreSurgery^*2^	M	121	0.14	0.45	0.88
	F	403	0.11	0.34	0.79
**Extension ROM**					
	nonparametric RIs				
	K-L class	n	LL	Me	UL
Healthy^*1^	All	120	-10	0	0
KOA-Outpatient	All	202	-15	-5	0
KOA-PreSurgery^*2^	All	545	-30	-5	0
**Flexion ROM**					
	nonparametric RIs				
	K-L class	n	LL	Me	UL
Healthy^*1^	All	120	120	150	160
KOA-Outpatient	All	202	95	130	150
	I・II	112	105	135	150
	III・IV	90	95	125	140
KOA-PreSurgery^*2^	All	545	90	125	150

Parameters indicated by (*1) represent the RI of healthy subjects, and those indicated by (*2) represent the RI of a presurgical patient

*RIs* Reference intervals, *LL* Lower limit, *Me* Median, *UL* Upper limit, *ROM* Range of motion, *K-L* Kellgren-Lawrence

Hence, the RIs for extensor muscle strength were 0.27–2.09 Nm/kg for men and 0.27–1.54 Nm/kg for women. The RIs for flexor muscle strength were 0.18–1.20 Nm/kg for men and 0.13–0.79 Nm/kg for women. The RIs of extension ROM were -15°–0°. The RIs of flexion ROM were 105°–150° for K-L grade I/II and 95°–140° for K-L grade III/IV.

## Discussion

The Guidelines for Physical Therapy in Japan [4] recommend physical therapy for patients with early KOA and decreased mobility. In clinical practice, knee muscle strength and ROM are measured to assess knee function in patients with KOA. To use these parameters objectively, reliable RIs should be established based on patients with KOA, including those with early-stage disease, and the factors contributing to their variability should be analyzed.

Our results revealed that knee extensor and flexor muscle strengths varied by sex, with decreased knee muscle strength observed in women. Similarly, Logerstedt et al. [20] compared knee muscle strength in patients with osteoarthritis by sex and reported that knee muscle strength in women was lower than that in men. In addition, knee muscle strength varied with BMI; higher BMI was associated with lower muscle strength. Andrews et al. [10] analyzed factors affecting muscle strength in healthy participants, and similar to our results, they inferred that body weight affected muscle strength values in patients with KOA.

Furthermore, only knee flexor strength decreased with age. Danneskiold-Samsøe et al. [9] reported that muscle strength decreased from 25 years in men and 40 years in women. Similarly, we speculated that knee flexion muscle strength in patients with KOA would decrease with age. However, knee extension and flexion ROM varied with the K-L grade; more severe KOA was associated with lower ROM. Ersoz and Ergun [21] discovered a negative correlation between ROM and KOA severity and reported that ROM was affected by the degree of compartment degeneration. Dickson et al. [22] also reported that KOA progression is influenced by quadriceps tendon stiffness. Therefore, as KOA progresses, ROM decreases owing to compartment degeneration and increased patellar tendon stiffness. Conversely, knee muscle strength did not decrease with KOA progression. Logerstedt et al. [20] compared knee muscle strength in healthy participants with that in patients with severe KOA and reported that the knee muscle strength in severe KOA patients was lower; however, associations were not investigated in the study. In contrast, Thomas et al. [23] reported that women with mild osteoarthritis did not present with quadriceps dysfunction, and Palmieri-Smith et al. [24] reported that KOA patients had decreased knee muscle strength compared with healthy participants; however, no difference was observed in muscle strength when K-L grades II, III, and IV were compared. Knee muscle strength is expected to intensify pain and thereby decreases muscle strength further as KOA progressed. However, Muraki et al. [25] reported that the severity of KOA did not affect the relationship between pain and loss of knee muscle strength. Therefore, muscle strength was lower than that in healthy participants but did not vary according to KOA severity.

We considered sex, age, BMI, and K-L grade as possible factors requiring subgrouping of test results in deriving their RIs. As presented in Table 3, by setting SDR ≥ 0.4 as the threshold for muscle strength values, partitioning by sex was required for extensor and flexor muscle strengths (SDRsex values = 0.652 and 0.567, respectively). In contrast, partitioning by the K-L grade was required for flexion ROM (SDRk-l value = 0.538). Furthermore, the muscle strength of the outpatients was intermediate between those of the healthy and preoperative patients. Pereira et al. [26] investigated normal knee extensor muscle strength in healthy elderly females and reported 40–60 percentile values of 1.30–1.43 Nm/kg, which differs greatly compared with its median value determined in this study for female KOA outpatients (0.72 Nm/kg with the RI of 0.27–1.54). Whereas it also differs from the meidan of the RI for extensor muscle strength we previously reported for preoperative female KOA patients (0.65 Nm/kg with the RI of 0.20–1.36) [11]. Therefore, RIs established in healthy adults and preoperative KOA patients may not be suitable for functional assessment of outpatients with mild to moderate KOA.

Reference values for ROM reported by the Japanese Society of Rehabilitation Medicine [17] are 130° for flexion ROM or 0° for extension ROM. However, we thought it necessary to determine the reference values as a reference interval (range), and hence we conducted a study for determining the RIs from preoperative KOA patients and healthy controls. Their RIs for knee flexion ROM were 90° − 150° and 120° − 160°, respectively, and for extension ROM were -30 − 0° and -10 − 0° [11]. In this study, we determined the RIs of KOA outpatients for flexion ROM as 105 − 150° (for K-L grade I/II) and 95 − 140° for (for K-L grade III/IV) and for extension ROM as -15 − 0° regardless of K-L grade. These RIs were in-between those of healthy controls and preoperative KOA patients. Obviously, the RIs for ROMs stratified by severity is necessary for functional assessment of outpatient KOA patients.

Clinically, we believe that using the RIs established by outpatients to evaluate the motor function of patients with KOA will help determine the indications for outpatient physical therapy. Furthermore, the use of RIs of knee joint flexion range of motion established according to the severity of KOA can be used as a guide to the effectiveness of outpatient physical therapy in preventing the progression of KOA. Therefore, we believe that the RIs established from outpatients with KOA can be used clinically by physicians and physical therapists in the field of musculoskeletal rehabilitation.

One limitation of this study was that the small sample size for males, which was due to stratifying the RI of muscle strength by sex. This was inevitable due to its overall low prevalence of KOA in males. Therefore, the UL and LL of the RI values for the males are not accurate. The sample size of male patients with KOA should be increased in future studies.

## Conclusions

As the number of patients with KOA increases, objective reference values are needed to assess knee function. By collaboration of eight orthopedic clinics in Japan, 202 patients with mild to moderate KOA were enrolled. RIs were determined separately by sex for extensor and flexor muscle strength, while RIs for flexion ROM were determined by the K-L grade. The ranges of RIs determined specifically for patients with mild to moderate KOA were in-between those of age-matched healthy controls and pre-surgical KOA patients, both of which we recently reported for use in physiotherapeutic management of KOA patients who underwent total knee arthroplasty [11]. The newly derived RIs will provide an objective benchmark for physiotherapy targeting highly prevalent outpatients with KOA.

## Data Availability

The datasets generated and/or analyzed during the current study are not publicly available due [To protect personal information.] but are available from the corresponding author on reasonable request.

## Abbreviations

KOA: Knee osteoarthritis
ROM: Range of motion
RIs: Reference intervals
SVs: Sources of variation
SD: Standard deviation
BMI: Body mass index
K-L: Kellgren–Lawrence
HHD: Handheld dynamometer
rp: Partial regression coefficient
ANOVA: Analysis of variance
SDsex: SD for sex
SDage: SD for age
SDbmi: SD for BMI
SDk-l: SD for the K-L classification
SDindiv: SD for between-individual variations
SDR: Standard deviation ratio
LL: Lower limit
UL: Upper limit
